# Impact of physical distancing measures against COVID-19 on contacts and mixing patterns: repeated cross-sectional surveys, the Netherlands, 2016–17, April 2020 and June 2020

**DOI:** 10.2807/1560-7917.ES.2021.26.8.2000994

**Published:** 2021-02-25

**Authors:** Jantien A Backer, Liesbeth Mollema, Eric RA Vos, Don Klinkenberg, Fiona RM van der Klis, Hester E de Melker, Susan van den Hof, Jacco Wallinga

**Affiliations:** 1 Centre for Infectious Disease Control, National Institute for Public Health and the Environment, Bilthoven, the Netherlands; 2 Department of Biomedical Data Sciences, Leiden University Medical Center, Leiden, the Netherlands

**Keywords:** COVID-19, SARS-CoV2, coronavirus, mixing patterns, physical distancing, contact survey

## Abstract

**Background:**

During the COVID-19 pandemic, many countries have implemented physical distancing measures to reduce transmission of SARS-CoV-2.

**Aim:**

To measure the actual reduction of contacts when physical distancing measures are implemented.

**Methods:**

A cross-sectional survey was carried out in the Netherlands in 2016–17, in which participants reported the number and age of their contacts the previous day. The survey was repeated among a subsample of the participants in April 2020, after strict physical distancing measures were implemented, and in an extended sample in June 2020, after some measures were relaxed.

**Results:**

The average number of community contacts per day was reduced from 14.9 (interquartile range (IQR): 4–20) in the 2016–17 survey to 3.5 (IQR: 0–4) after strict physical distancing measures were implemented, and rebounded to 8.8 (IQR: 1–10) after some measures were relaxed. All age groups restricted their community contacts to at most 5, on average, after strict physical distancing measures were implemented. In children, the number of community contacts reverted to baseline levels after measures were eased, while individuals aged 70 years and older had less than half their baseline levels.

**Conclusion:**

Strict physical distancing measures greatly reduced overall contact numbers, which likely contributed to curbing the first wave of the COVID-19 epidemic in the Netherlands. However, age groups reacted differently when measures were relaxed, with children reverting to normal contact numbers and elderly individuals maintaining restricted contact numbers. These findings offer guidance for age-targeted measures in future waves of the pandemic.

## Introduction

Since the beginning of 2020, the severe acute respiratory syndrome coronavirus 2 (SARS-CoV-2) that causes coronavirus disease (COVID-19) has rapidly spread around the world. Most COVID-19 cases experience mild symptoms, but elderly individuals and those with comorbidities are at higher risk of severe acute respiratory disease [1]. Hospitals have been confronted with very high numbers of patients, often in excess of intensive care capacity. Countries have implemented control measures, including increased hygiene, travel restrictions, case finding, contact tracing and physical distancing. Specific physical distancing measures differ between countries and regions; however, their overall aim is to reduce the number of contacts in the population, thus preventing the transmission of infection. The impact that physical distancing measures have on reducing contacts in the population, and how reduction of contacts may vary by age group, is rarely quantified.

A variety of approaches are used to measure behavioural changes. Mobile telephone data provided by telecom companies are used to measure changes in mobility patterns [2,3]; similarly, smartphones’ location history can be tracked with apps [4]. These anonymised and aggregated mobility patterns can suggest contact pattern changes in the population at large. To obtain direct and detailed information on contact numbers and patterns, cross-sectional studies are conducted in which participants report their age and sex, as well as the age and sex of all persons they had contact with on a given day [5,6]. In the first months of the COVID-19 pandemic, contact surveys were used to quantify the reduction in the number of contacts associated with physical distancing measures in Shanghai and Wuhan, China, estimated at 88% and 86%, respectively [7], and in the United Kingdom (UK), among the adult population, estimated at 74% [8]. One challenge of the contact survey approach is obtaining a reliable baseline measurement before physical distancing measures are implemented. In the Wuhan study, participants needed to recall their number of contacts on a regular weekday at the end of 2019. In the UK study, the baseline was provided by a similar study conducted 13 years ago among a different representative UK study population [5].

We present two large contact surveys conducted in the Netherlands in April and June 2020. The participants were recruited from a large nationwide sample of the Dutch population who had participated in an earlier cross-sectional survey in 2016–17 [9]. The contact questionnaire was nearly identical in all three surveys, which allowed us to use the earlier survey as a baseline measurement.

By 16 March 2020, the Netherlands had imposed strict physical distancing measures to control the spread of COVID-19, including closing daycare centres, schools, universities, cafes, pubs, restaurants, theatres, cinemas and sport clubs, as well as cancelling events with more than 10 persons attending. The advice to citizens was to work from home whenever possible and to maintain 1.5 m distance from others outside their household. The first 2020 survey was conducted a few weeks after these measures were implemented. By 1 June 2020, most of the strict physical distancing measures were relaxed, except for the recommendations to work from home and keep 1.5 m distance from others. Primary schools and daycare centres had re-opened and operated at full capacity, and secondary schools, cafes, pubs, restaurants, theatres and cinemas had re-opened and operated at a reduced capacity. A few weeks after this relaxation of measures, the second 2020 survey was conducted.

By comparing the survey results, we were able to determine the physical distancing measures’ impact on the number of contacts made in the community (i.e. outside the household) and could distinguish between different age groups, sexes, household sizes and days of the week. We also assessed how the measures affected the total number of contacts, including contact with household members, and the age-specific mixing patterns.

## Methods

From February 2016 to October 2017, a cross-sectional, sero-epidemiological study was conducted in a sample of the Dutch population aged between 0 to 89 years old [9]; henceforth, this is referred to as the ‘baseline survey’. Participants were randomly selected from the Dutch population registry using a two-stage cluster design. Infants < 1 year old, people living in areas with low vaccination coverage and people with a migration background were oversampled in this survey. The study consisted of an extensive questionnaire that was filled out by parents or guardians for participants < 15 years old. It included questions regarding the participants’ age and sex, the age and sex of their household members, the total number of unique people they had contact with outside their household the previous day and which day of the week this was, i.e. Monday through Sunday, hereafter referred to as the ‘contact day’. Contacts’ ages were reported using the following age groups: 0–4, 5–9, 10–19, 20–29, 30–39, 40–49, 50–59, 60–69, 70–79, 80–89 and ≥ 90. In the questionnaire, examples of what constitutes contact are given, such as talking face-to-face, touching, kissing someone or playing sports with someone.

Of the 8,179 baseline survey participants, 6,102 were invited to participate in the follow-up study on 26 March 2020, referred to herein as the ‘April 2020 survey’ [10]. In total, we received 3,168 questionnaire responses for the April 2020 survey. Of these participants, 2,754 participated in the final survey, referred to herein as the ‘June 2020 survey’ [11]. The June 2020 survey was supplemented with 4,496 new participants from a large population-based sample of 27,053 randomly selected Dutch citizens across all municipalities, bringing the total number of participants for the June 2020 survey to 7,250.

In order to obtain a representative study population, we omitted participants from the areas with low vaccination coverage that were oversampled and the oversampled infants < 1 year old from the baseline survey, so that the fraction of infants in the study population reflected that of the 2017 Dutch population. We also excluded participants that did not report their household composition, as well as those that did not report any contacts and omitted the contact day. Finally, participants reporting more than 100 contacts per day were excluded from the analysis, as it was deemed unrealistic to have had so many face-to-face conversations in one day.

The questionnaire used in both 2020 surveys was identical to that of the baseline survey, except for two questions. We added one question that asked whether participants had made any contacts outside of their household (the baseline survey directly asked to list the contacts) and, in the June 2020 survey, there was one further question asking how many contacts in each age group occurred within 1.5 m or beyond 1.5 m.

We analysed the contact surveys by comparing the number of contacts in the community per participant, stratified by several characteristics: age, sex, household size and contact day. We combined the two oldest age groups (80–89 and ≥ 90) into ≥ 80 years for the analysis. For the June 2020 survey, we studied the fraction of close contacts (within 1.5 m) by age group. We restricted this part of the analysis to community contacts, i.e. contacts made with non-household members, because contact with household members was not reported.

For the following part of the analysis, we used the household composition as a proxy for household contacts. By adding these to the reported community contacts, we obtained the total number of contacts in the population. We estimated age-stratified contact matrices that contain the number of contacts made between and within age groups, using an approach that accounts for reciprocity of contacts between different age groups [12] and age-specific population size data for the Netherlands on 1 January 2017 and 1 January 2019 [13]. To check the effect of enforcing reciprocity between contacts, we compared the estimated and observed mean number of contacts per participant. We characterised the mixing pattern of the age-specific contacts by the disassortativeness index [14], which indicates assortative mixing for values of 0 and random mixing for values of 1. We characterised the ‘effective number’ of age-specific contacts by the largest eigenvalue of the contact matrix [15]. All analyses were done using R version 3.6.0 [16].

### Ethics approval and consent to participate

The Medical Research Ethics Committees United (MEC-U; R20.022) approved the research protocol ‘Third population-based immune surveillance study used for the evaluation of immunity against SARS-CoV-2 (PIENTER Corona)’ and written informed consent was obtained from all adult participants and parents or legal guardians of minors included in the study.

## Results

### Characteristics of the study population

In total, 5,066 baseline survey participants, 2,069 April 2020 survey participants and 6,300 June 2020 survey participants were included for analysis.

**Figure 1:**
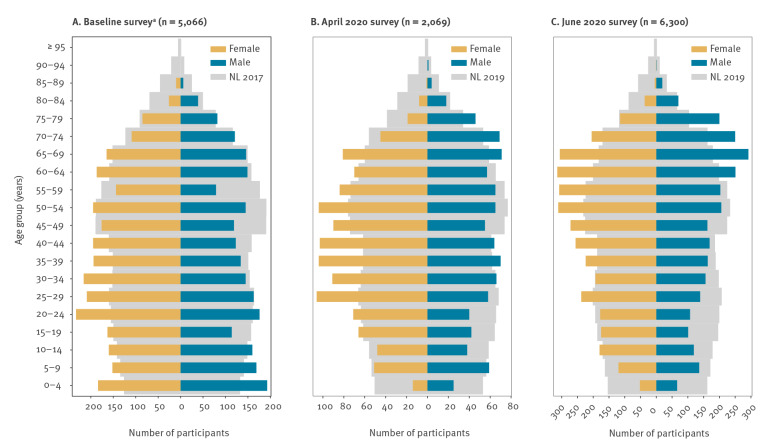
Age and sex of survey participants, the Netherlands, (A) baseline survey 2016–17^a^, (B) April 2020 and (C) June 2020

The composition of the survey population by age and sex should reflect the Dutch population (Figure 1), but the older age groups were overrepresented in the 2020 surveys. The mean age was 37 years (range: 0–88 years) in the baseline survey, 42 years (range: 3–90 years) in the April 2020 survey and 46 years (range: 1–90 years) in the June 2020 survey, whereas the mean age of the Dutch population is 41 years. In all surveys, each age group consisted of more than 100 participants, except for the ≥ 80 years age group in the baseline survey and the 0–4 years and ≥ 80 age groups in the April 2020 survey (Table 1). As expected from the household size distribution in the Netherlands [17], participants lived mostly in two-person households, followed by four-person households. Although the April 2020 survey population contained relatively few single-person households, the reported average household size was similar across all surveys (2.8 persons for the baseline survey, 3.0 for the April 2020 survey, 2.9 for the June 2020 survey) and was in line with the expected average household size of 2.8 persons. There were at least 79 participants per day of the week for each survey.

NL: population of the Netherlands.

^a^ The baseline survey took place from February 2016 to October 2017.

The baseline, April 2020 and June 2020 surveys are compared with the Dutch population in 2017, 2019 and 2019, respectively. Please note the different scales of the x axes.

**Table 1:** Characteristics of survey participants, the Netherlands, baseline survey 2016–17^a^, April 2020 and June 2020

Characteristics	Baseline survey^a^		April 2020 survey		June 2020 survey	
	n	%	n	%	n	%
**Total**	**5,066**	**100**	**2,069**	**100**	**6,300**	**10**
**Age group (years)**						
0–4	377	7.4	39	1.9	118	1.9
5–9	321	6.3	110	5.3	256	4.1
10–19	597	11.8	194	9.4	575	9.1
20–29	781	15.4	275	13.3	662	10.5
30–39	689	13.6	331	16.0	736	11.7
40–49	613	12.1	312	15.1	859	13.6
50–59	563	11.1	318	15.4	1,028	16.3
60–69	647	12.8	279	13.5	1,163	18.5
70–79	397	7.8	179	8.7	770	12.2
≥ 80	81	1.6	32	1.5	133	2.1
**Sex**						
Female	2,803	55.3	1,156	55.9	3,490	55.4
Male	2,263	44.7	913	44.1	2,810	44.6
**Household size^b^**						
1	882	17.4	168	8.1	700	11.1
2	1,844	36.4	727	35.1	2,499	39.7
3	667	13.2	359	17.4	902	14.3
4	1,068	21.1	562	27.2	1,496	23.7
5	451	8.9	197	9.5	543	8.6
≥ 6	154	3.0	56	2.7	160	2.5
**Contact day**						
Monday	918	18.1	521	25.2	1,113	17.7
Tuesday	831	16.4	546	26.4	1,047	16.6
Wednesday	517	10.2	368	17.8	1,004	15.9
Thursday	264	5.2	205	9.9	672	10.7
Friday	428	8.4	102	4.9	367	5.8
Saturday	683	13.5	79	3.8	746	11.8
Sunday	980	19.3	246	11.9	1,348	21.4
Missing	445	8.8	2	0.1	3	0.0

^a^ The baseline survey took place from February 2016 to October 2017.

^b^ Households in the Netherlands consist of one (38%), two (33%), three (12%), four (12%) and more than 5 (5%) persons [17], leading to the following expected household size distribution per participant: one (17%), two (31%), three (17%), four (23%) or more than five (12%) persons.

### Reduction in the mean number of community contacts

The percentage of participants who did not report any community contacts on a single day increased from 5% in the baseline survey to 42% in the April 2020 survey, and decreased again to 22% in the June 2020 survey. The average number of community contacts a participant reported per day decreased from 14.9 (interquartile range (IQR): 4–20) in the baseline survey to 3.5 (IQR: 0–4) in the April 2020 survey, and rebounded to 8.8 (IQR: 1–10) in the June 2020 survey (Table 2). Compared with the baseline survey, the number of community contacts reduced by 76% and 41% in the April 2020 and June 2020 surveys, respectively.

**Table 2:** Number of community contacts per survey participant, the Netherlands, baseline survey 2016–17^a^ (n = 5,066), April 2020 (n = 2,069) and June 2020 (n = 6,300)

Characteristics	Baseline survey^a^		April 2020 survey		June 2020 survey	
	Mean	IQR	Mean	IQR	Mean	IQR
**Total**	**14.9**	**4–20**	**3.5**	**0–4**	**8.8**	**1–10**
**Age group (years)**						
0–4	13.8	3–19	3.7	0–4	18.0	3–26
5–9	22.1	6–33	2.1	0–3	27.1	6–39
10–19	22.5	7–33	2.9	0–4	14.3	1–24
20–29	16.6	5–22	3.6	0–5	9.4	1–11
30–39	14.3	5–18	4.2	0–6	9.7	2–12
40–49	15.3	4–20	4.8	0–5	9.3	2–11
50–59	13.0	4–16	4.7	0–6	7.1	1–9
60–69	9.7	3–11	2.2	0–3	5.9	0–6
70–79	7.8	2–9	1.8	0–2	2.7	0–4
≥ 80	7.9	2–8	0.7	0–1	3.2	0–4
**Sex**						
Female	14.3	4–19	3.4	0–4	8.9	1–10
Male	15.6	4–22	3.7	0–4	8.7	1–10
**Household size^b^**						
1	12.7	3–16	3.1	0–3	5.2	0–6
2	13.2	3–17	3.0	0–3	5.8	0–7
3	13.9	4–19	4.5	0–5	10.1	1–12
4	17.4	5–25	3.7	0–4	12.1	2–15
5	19.2	6–29	3.7	0–5	13.5	2–18
≥ 6	20.5	6–30	3.1	0–4	16.5	2–24
**Contact day**						
Monday	16.0	4–22	3.5	0–4	10.4	1–12
Tuesday	17.1	5–25	3.5	0–4	10.0	1–12
Wednesday	15.8	5–21	3.9	0–4	9.3	1–11
Thursday	17.0	4–24	4.3	0–5	9.4	1–11
Friday	16.3	4–22	3.6	0–4	13.6	2–16
Saturday	12.9	4–17	4.2	0–4	8.1	2–9
Sunday	10.1	3–12	2.4	0–3	4.8	0–5
Missing	18.1	5–25	3.0	3–3	21.0	2–31

IQR: interquartile range.

^a^ The baseline survey took place from February 2016 to October 2017.

^b^ Persons per household.

The relative change in mean number of community contacts compared with the baseline survey is shown in shades of green (for reduction) and red (for increase).

**Figure 2:**
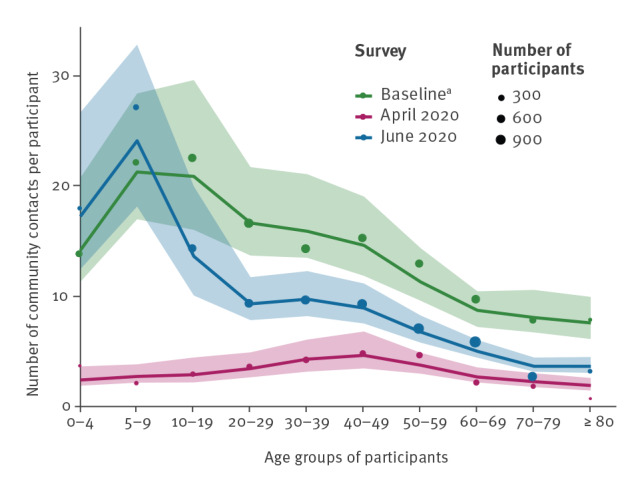
Number of community contacts per survey participant, by age group, the Netherlands, baseline survey 2016–17^a^ (n = 5,066), April 2020 (n = 2,069) and June 2020 (n = 6,300)

^a^ The baseline survey took place from February 2016 to October 2017.

In the baseline survey, participants aged 10 to 19 years had the highest number of community contacts, and this number gradually declined as age increased. In contrast, the number of community contacts was more similar across the different age categories in the April 2020 survey, with the 30–59 years age group reporting the highest contact numbers (Figure 2). The reduction in the number of contacts was greatest for participants aged 5 to 9 years, 90%, and lowest for participants aged 50 to 59 years, 64% (Table 2). In June 2020, after strict physical distancing measures had been relaxed, contact numbers increased among all age groups. Compared with the baseline survey, the two oldest age groups (70–79 years and ≥ 80 years) still reduced their contacts by 65% and 59%, respectively, but the two youngest age groups (0–4 years and 5–9 years) increased their number of contacts by 30% and 23%, respectively.

Shown are the estimated mean numbers of contacts after accounting for reciprocity in contacts (line) with the corresponding 95% credible interval (shaded area) and the observed means (size of dots, scaled to number of participants).

The reduction in the mean number of contacts was similar for male and female participants in both 2020 surveys. The number of community contacts increased with household size in the baseline survey, whereas this number was similar regardless of household size in the April 2020 survey. In the June 2020 survey, the number of community contacts increased for all household sizes, albeit to a lesser extent in one- and two-person households. This is likely because of the high proportion of elderly people who live in smaller households (Supplement S1.1). In the baseline survey, a higher number of contacts were reported on weekdays than on weekends. This distinction nearly disappeared during the strict physical distancing measures, but re-appeared after measures were relaxed.

### Contacts within or beyond 1.5 m distance

**Figure 3:**
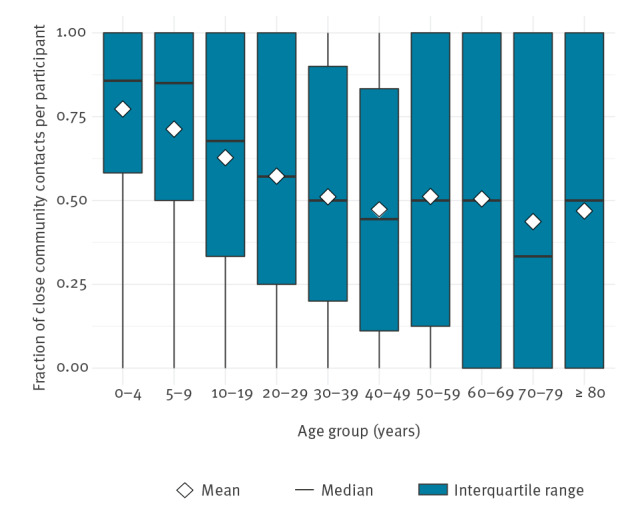
Fraction of close community contacts per survey participant, by age group, the Netherlands, June 2020 (n = 6,300)

In the June 2020 survey, survey participants were asked to include whether contacts occurred within or beyond 1.5 m distance. On average, 53% of the community contacts per participant that were reported occurred within 1.5 m, ranging from 44% for the 70–79 years age group to 77% for the 0–4 years age group (Figure 3). These close contacts outside the household occur mainly among children, and between 0–4 year olds and 50–79 year olds (Supplement S1.2).

Close contacts were defined as occurring within 1.5 m.

### Comparison of mixing patterns

In estimating the contact rates between age groups, reciprocity between contacts was explicitly taken into account. The observed and estimated mean numbers of community contacts per participant are in agreement (Figure 2), showing consistency in reporting of contacts between the age groups.

**Figure 4:**
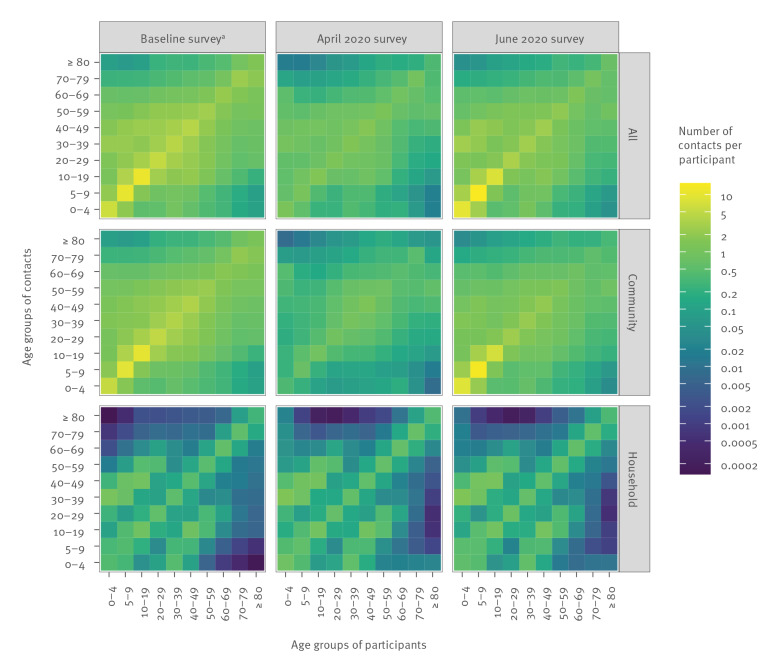
Estimated contact matrices for all contacts, contacts in the community and contacts with household members, the Netherlands, baseline survey 2016–17^a^ (n = 5,066), April 2020 (n = 2,069) and June 2020 (n = 6,300)

^a^ The baseline survey took place from February 2016 to October 2017.

The contact matrices for all surveys and contact types are shown in Figure 4 (Supplement S2). The contact matrices for household members illustrate that participants generally live with persons in their own age group and with their children or parents (i.e. 30 years younger or older); this is apparent in all surveys, as the household composition remains fairly constant over time. The matrices for contacts in the community indicate that the younger age groups had fewer contacts with all other age groups in the April 2020 survey compared with the baseline survey. Most community contacts in the April 2020 survey were among people in the working-age age groups and between elderly people (≥ 80 years old) and adults (40–69 years old) who might be healthcare workers or informal caregivers, including family members living in a different household. In the June 2020 survey, the community contact pattern was restored to the baseline pattern, but the absolute values of the contact numbers were smaller.

Taken together, the household and community contact patterns reveal that age-specific mixing did not change much between the baseline and the April 2020 surveys (disassortativeness index was 0.50 for the baseline survey and 0.52 for the April 2020 survey). For the June 2020 survey, however, the disassortativeness index was 0.66, indicating that during this period mixing shifted slightly from within the same age groups to between different age groups, which could enable viruses to spread more easily in the population. This change in mixing patterns was reflected in the effective number of contacts per person (i.e. the largest eigenvalue of the contact matrix), which decreased from 18 (95% credible interval (CrI): 15–24) in the baseline survey to 5.6 (95% CrI: 4.7–6.5) in the April 2020 survey, an average reduction of 69% (95% CrI: 58%–77%), and reverted to 18 (95% CrI: 14–27) in the June 2020 survey.

## Discussion

In the Netherlands, strict physical distancing measures to control the spread of SARS-CoV-2 came into effect on 16 March 2020. Three weeks after their implementation, the number of intensive care beds occupied by COVID-19 patients peaked above 1,300, and then declined to below 100 by mid June 2020 [18], when most measures had been relaxed to some degree. We show that in comparison with the baseline survey, strict physical distancing measures reduced community contacts by 76% in April 2020, while the reduction was 41% in June 2020, after the measures were relaxed.

The sampling scheme for inviting participants from the Dutch population was designed to obtain a representative study population. To assess the representativeness of the survey participants, a few potential limitations need to be addressed. First, not all of those who were invited to the survey participated, causing a potential for selection bias. Second, there are differences in participants’ characteristics and their interactions with contacts between the surveys; for instance, age, sex, household size and contact day. We determined the weighted average number of contacts to account for these differences (Supplement S1.3) and found that only the participant age altered the unweighted averages by ca 10%. This does not affect the results, as all of them are stratified by age. Third, the 2020 surveys were carried out over 1 month (April and June), whereas the baseline survey was conducted over a period of almost 2 years. Because contact patterns change little throughout the course of a year (Supplement S1.4 and [19]), we do not expect this to substantially affect the estimated reduction. Finally, the surveys consisted of different but overlapping study populations. To check whether this had any effect on the results, we repeated the analysis on 1,739 participants that participated in all surveys. Results showed that—although baseline levels were a bit higher—trends and reductions were similar to the main analysis (Supplement S1.5).

During strict physical distancing, the number of community contacts was drastically reduced in all age groups. When these measures were relaxed, people aged 70 years and older largely kept their contacts at a reduced level, while children less than 10 years old had a number of contacts similar to before the measures were implemented. Moreover, the majority of the children’s contacts occurred within 1.5 m, whereas in the rest of the population around half of the contacts were in close range. The number of contacts of children less than 10 years old is more than twice the population mean number of contacts (Table 2), while the incidence of reported infections in this age group is less than half the population mean incidence [20]. This supports findings that children play a minor role in the epidemic, as was also found in age-specific seroprevalence studies [21-23].

The reduction in the number of contacts associated with strict physical distancing measures in the Netherlands was smaller than the reductions of 88% and 86% observed in Shanghai and Wuhan, China [7], most likely because the measures in the Netherlands were less stringent than those in both Chinese cities. In the UK, a 74% reduction in the number of all contacts among adults (≥ 18 years old) has been reported [8]. To compare this with our results, we calculated the total number of contacts, including community and household contacts, for the participants ≥ 18 years old in our surveys, and found a reduction of 72%. At the time of these two studies, the control measures in the UK and the Netherlands ranked similarly according to the stringency index [24], which seems to have led to a similar contact reduction in the adult population.

Compared with the baseline, the effective number of contacts per person was found to be reduced by 69% in the April 2020 survey and by 0% in the June 2020 survey. This number would be proportional to the reproduction number (i.e. the number of secondary infections caused by a single infectious person in the population) under three conditions. First, the definition of contact (having a face-to-face conversation or physical contact) would need to be a good proxy measure for at-risk contact events where SARS-CoV-2 can be transmitted. This condition is likely met, as the virus transmits through similar routes as influenza virus, i.e. droplets, fomites, aerosols and contaminated surfaces, for which this is a validated approach [25]. Second, contacts should have been made in a similar fashion in the different surveys. Conversational contacts during the pandemic may very well have taken place at a greater distance or with a face mask; therefore, the reproduction number may have been further reduced, even more than by the reduction in the effective number of contacts. Third, all age groups would need to be equally susceptible and infectious. As evidence accrues that children are less infectious or less susceptible [7,26-28], and as they have the largest number of contacts in the baseline survey, the reduction of the reproduction number would consequently be less than the reduction in the effective number of contacts of 69% in the April 2020 survey. After the relaxation of strict physical distancing measures, contact mixing shifted even more to younger age groups. Because of their limited role in transmission, the reduction of the effective reproduction number should exceed the reduction in the effective number of contacts of 0% in June 2020. Also, general hygiene measures and the use of face masks will have led to a reduction of the reproduction number, but this effect is not captured in the contact matrices.

The results of this study can immediately be applied to the public health response and management of the COVID-19 pandemic. The estimated contact reduction is applicable to other countries and regions with similar control measures. Combined with appropriate susceptibility and infectiousness profiles, the estimated age-specific contact matrices are useful for conducting scenario analyses with age-structured transmission models of COVID-19, to project the future course of the epidemic with or without physical distancing measures [29-31]. We believe that contact surveys such as these can help to inform and guide infection control measures. Repeating the contact survey at regular intervals will capture the number of contacts made over time and the fraction of close contacts. This can support assessment of the impact of renewed physical distancing measures, as well as changes in compliance.

## Supplementary Data

233000 Supplement S2

## Supplementary Data

696000 Supplement S1
