# Supplementary material for: Impact of physical distancing measures against COVID-19 on contacts and mixing patterns: repeated cross-sectional surveys, the Netherlands, 2016–17, April 2020 and June 2020
Source: Euro Surveill. 2021 Feb 25;26(8):2000994. doi: 10.2807/1560-7917.ES.2021.26.8.2000994 (PMC7908067; doi:10.2807/1560-7917.ES.2021.26.8.2000994)
Supplement: Supplement S2 [file 20-00994_BACKER_Supplement2.pdf]

This supplementary material is hosted by *Eurosurveillance* as supporting information alongside the article 'Impact of physical distancing measures against COVID-19 on contacts and mixing patterns: repeated cross-sectional surveys, the Netherlands, 2016-17, April 2020 and June 2020', on behalf of the authors, who remain responsible for the accuracy and appropriateness of the content. The same standards for ethics, copyright, attributions and permissions as for the article apply. Supplements are not edited by *Eurosurveillance* and the journal is not responsible for the maintenance of any links or email addresses provided therein.

S2. Data of contact matrices

survey	contact_type	part_age	cont_age	m_est
baseline	all	[0,5)	[0,5)	6.139521635385062
baseline	all	[0,5)	[5,10)	2.0527165928673994
baseline	all	[0,5)	[10,20)	1.175997998243935
baseline	all	[0,5)	[20,30)	1.364982706773537
baseline	all	[0,5)	[30,40)	2.2240427117384405
baseline	all	[0,5)	[40,50)	1.5889823150802727
baseline	all	[0,5)	[50,60)	1.0548466633926246
baseline	all	[0,5)	[60,70)	0.67532922983989
baseline	all	[0,5)	[70,80)	0.23854694625315218
baseline	all	[0,5)	[80,Inf]	0.08774812816606976
baseline	all	[5,10)	[0,5)	1.92697166301877
baseline	all	[5,10)	[5,10)	10.504839348460953
baseline	all	[5,10)	[10,20)	3.6045354064172415
baseline	all	[5,10)	[20,30)	1.9542018293314807
baseline	all	[5,10)	[30,40)	2.202378931284297
baseline	all	[5,10)	[40,50)	2.1516106498031857
baseline	all	[5,10)	[50,60)	1.1610568208259353
baseline	all	[5,10)	[60,70)	0.6312683395015201
baseline	all	[5,10)	[70,80)	0.2533635834408248
baseline	all	[5,10)	[80,Inf]	0.06958862088202837
baseline	all	[10,20)	[0,5)	0.5089127775157799
baseline	all	[10,20)	[5,10)	1.661601516803311
baseline	all	[10,20)	[10,20)	11.19323165333237
baseline	all	[10,20)	[20,30)	2.9661845764299835

baseline	all	[10,20)	[30,40)	1.9023619378235366
baseline	all	[10,20)	[40,50)	2.487524570869481
baseline	all	[10,20)	[50,60)	1.6280378588748066
baseline	all	[10,20)	[60,70)	0.6610149672749319
baseline	all	[10,20)	[70,80)	0.2583349890120911
baseline	all	[10,20)	[80,Inf]	0.08542946453836944
baseline	all	[20,30)	[0,5)	0.5521773622197372
baseline	all	[20,30)	[5,10)	0.8421011091734943
baseline	all	[20,30)	[10,20)	2.7727429593223625
baseline	all	[20,30)	[20,30)	5.284430071050166
baseline	all	[20,30)	[30,40)	2.702265307635128
baseline	all	[20,30)	[40,50)	2.43894333022775
baseline	all	[20,30)	[50,60)	2.02359873081602
baseline	all	[20,30)	[60,70)	0.886689654288011
baseline	all	[20,30)	[70,80)	0.34632060383679847
baseline	all	[20,30)	[80,Inf]	0.22876797379690542
baseline	all	[30,40)	[0,5)	0.9462173536707417
baseline	all	[30,40)	[5,10)	0.9981168126264318
baseline	all	[30,40)	[10,20)	1.8702521868868922
baseline	all	[30,40)	[20,30)	2.8419964309002173
baseline	all	[30,40)	[30,40)	4.143537717960831
baseline	all	[30,40)	[40,50)	2.9687596984561706
baseline	all	[30,40)	[50,60)	2.0021661690617973
baseline	all	[30,40)	[60,70)	1.0386437286892038
baseline	all	[30,40)	[70,80)	0.42258875215409086
baseline	all	[30,40)	[80,Inf]	0.26275265433434003
baseline	all	[40,50)	[0,5)	0.5881973964185363
baseline	all	[40,50)	[5,10)	0.8484156143985718
baseline	all	[40,50)	[10,20)	2.127819975772693
baseline	all	[40,50)	[20,30)	2.2318070785360686
baseline	all	[40,50)	[30,40)	2.583084584033303

baseline	all	[40,50)	[40,50)	4.0598661736153545
baseline	all	[40,50)	[50,60)	2.2355661616994307
baseline	all	[40,50)	[60,70)	1.059590165511839
baseline	all	[40,50)	[70,80)	0.4996273722515741
baseline	all	[40,50)	[80,Inf]	0.24827546907714237
baseline	all	[50,60)	[0,5)	0.3720467931635602
baseline	all	[50,60)	[5,10)	0.4362126465141529
baseline	all	[50,60)	[10,20)	1.3268884294375678
baseline	all	[50,60)	[20,30)	1.7643273256527996
baseline	all	[50,60)	[30,40)	1.6598401761921713
baseline	all	[50,60)	[40,50)	2.1300556682278553
baseline	all	[50,60)	[50,60)	2.7446057150540275
baseline	all	[50,60)	[60,70)	1.3302552326398727
baseline	all	[50,60)	[70,80)	0.5796213551064978
baseline	all	[50,60)	[80,Inf]	0.3405722962771107
baseline	all	[60,70)	[0,5)	0.2826752058499303
baseline	all	[60,70)	[5,10)	0.2814714600639131
baseline	all	[60,70)	[10,20)	0.6393681975131799
baseline	all	[60,70)	[20,30)	0.9174847454335503
baseline	all	[60,70)	[30,40)	1.0218833408928012
baseline	all	[60,70)	[40,50)	1.198154121849265
baseline	all	[60,70)	[50,60)	1.5787364495766136
baseline	all	[60,70)	[60,70)	2.199995059391792
baseline	all	[60,70)	[70,80)	1.0280006598918592
baseline	all	[60,70)	[80,Inf]	0.43441584431871605
baseline	all	[70,80)	[0,5)	0.15081485263202044
baseline	all	[70,80)	[5,10)	0.17063509458401244
baseline	all	[70,80)	[10,20)	0.37741407313822284
baseline	all	[70,80)	[20,30)	0.5412414543671902
baseline	all	[70,80)	[30,40)	0.6279731562455445
baseline	all	[70,80)	[40,50)	0.8533205976883831

baseline	all	[70,80)	[50,60)	1.0389815413996486	
baseline	all	[70,80)	[60,70)	1.5527286546170018	
baseline	all	[70,80)	[70,80)	2.4740919069515948	
baseline	all	[70,80)	[80,Inf]	0.9990422350832439	
baseline	all	[80,Inf]	[0,5)	0.10014661285125653	
baseline	all	[80,Inf]	[5,10)	0.08460601833047882	
baseline	all	[80,Inf]	[10,20)	0.2253129686980694	
baseline	all	[80,Inf]	[20,30)	0.6454249318067132	
baseline	all	[80,Inf]	[30,40)	0.7048926991262647	
baseline	all	[80,Inf]	[40,50)	0.7654974328652318	
baseline	all	[80,Inf]	[50,60)	1.1020500223634009	
baseline	all	[80,Inf]	[60,70)	1.1845361749416363	
baseline	all	[80,Inf]	[70,80)	1.803649485947723	
baseline	all	[80,Inf]	[80,Inf]	1.4572052689113548	
baseline	community		[0,5)	[0,5)	5.431546880006931
baseline	community		[0,5)	[5,10)	1.7757184974779268
baseline	community		[0,5)	[10,20)	1.16139251024841
baseline	community		[0,5)	[20,30)	1.1090859430458224
baseline	community		[0,5)	[30,40)	1.4297316584824469
baseline	community		[0,5)	[40,50)	1.2554718803223726
baseline	community		[0,5)	[50,60)	1.0017166414257557
baseline	community		[0,5)	[60,70)	0.6525994581498071
baseline	community		[0,5)	[70,80)	0.241818158324518
baseline	community		[0,5)	[80,Inf]	0.085020556804826
baseline	community		[5,10)	[0,5)	1.6669384621544687
baseline	community		[5,10)	[5,10)	9.120774264458923
baseline	community		[5,10)	[10,20)	3.4911565538358924
baseline	community		[5,10)	[20,30)	1.8142515390421365
baseline	community		[5,10)	[30,40)	1.62869342361797
baseline	community		[5,10)	[40,50)	1.544594304991439
baseline	community		[5,10)	[50,60)	1.0268226282910429

baseline	community	[5,10)	[60,70)	0.6074364619049684
baseline	community	[5,10)	[70,80)	0.2512163173536992
baseline	community	[5,10)	[80,Inf]	0.07351803401031781
baseline	community	[10,20)	[0,5)	0.502592816743358
baseline	community	[10,20)	[5,10)	1.6093347895506156
baseline	community	[10,20)	[10,20)	9.971286058283768
baseline	community	[10,20)	[20,30)	2.942494829691994
baseline	community	[10,20)	[30,40)	1.7280153420885396
baseline	community	[10,20)	[40,50)	1.8176328098813872
baseline	community	[10,20)	[50,60)	1.2772294539808002
baseline	community	[10,20)	[60,70)	0.6245425065639117
baseline	community	[10,20)	[70,80)	0.2622873926561113
baseline	community	[10,20)	[80,Inf]	0.09141463011667969
baseline	community	[20,30)	[0,5)	0.44865893845256927
baseline	community	[20,30)	[5,10)	0.7817943475456287
baseline	community	[20,30)	[10,20)	2.750598087200861
baseline	community	[20,30)	[20,30)	4.869331858086486
baseline	community	[20,30)	[30,40)	2.572129846636442
baseline	community	[20,30)	[40,50)	2.166379158537687
baseline	community	[20,30)	[50,60)	1.6642495080364075
baseline	community	[20,30)	[60,70)	0.8036170842103314
baseline	community	[20,30)	[70,80)	0.3465468577084065
baseline	community	[20,30)	[80,Inf]	0.2102016420173198
baseline	community	[30,40)	[0,5)	0.6082766430072056
baseline	community	[30,40)	[5,10)	0.7381229176110249
baseline	community	[30,40)	[10,20)	1.6988469179641115
baseline	community	[30,40)	[20,30)	2.7051316902295084
baseline	community	[30,40)	[30,40)	3.7858328716476906
baseline	community	[30,40)	[40,50)	2.785329928151861
baseline	community	[30,40)	[50,60)	1.8786069793452755
baseline	community	[30,40)	[60,70)	0.9826090944779176

baseline	community	[30,40)	[70,80)	0.4283181817964187
baseline	community	[30,40)	[80,Inf]	0.25070171572558303
baseline	community	[40,50)	[0,5)	0.4647419174268971
baseline	community	[40,50)	[5,10)	0.6090592761094537
baseline	community	[40,50)	[10,20)	1.5547919860667434
baseline	community	[40,50)	[20,30)	1.9823912006606779
baseline	community	[40,50)	[30,40)	2.423483788626891
baseline	community	[40,50)	[40,50)	3.651824434401357
baseline	community	[40,50)	[50,60)	2.0985086620947224
baseline	community	[40,50)	[60,70)	1.0463616521393935
baseline	community	[40,50)	[70,80)	0.5020295792722752
baseline	community	[40,50)	[80,Inf]	0.24959129054171622
baseline	community	[50,60)	[0,5)	0.3533071995522148
baseline	community	[50,60)	[5,10)	0.38578049513300555
baseline	community	[50,60)	[10,20)	1.0409746024661868
baseline	community	[50,60)	[20,30)	1.4510208071446586
baseline	community	[50,60)	[30,40)	1.557405477104481
baseline	community	[50,60)	[40,50)	1.9994677844347233
baseline	community	[50,60)	[50,60)	2.3254011613690295
baseline	community	[50,60)	[60,70)	1.235040652586994
baseline	community	[50,60)	[70,80)	0.58925404827306
baseline	community	[50,60)	[80,Inf]	0.3328431174670522
baseline	community	[60,70)	[0,5)	0.2731609556479113
baseline	community	[60,70)	[5,10)	0.270845004132822
baseline	community	[60,70)	[10,20)	0.6040887608290078
baseline	community	[60,70)	[20,30)	0.8315253068437565
baseline	community	[60,70)	[30,40)	0.9667516978530365
baseline	community	[60,70)	[40,50)	1.1831944114603787
baseline	community	[60,70)	[50,60)	1.4657366974417108
baseline	community	[60,70)	[60,70)	1.7155453613420382
baseline	community	[60,70)	[70,80)	0.9458226608420033

baseline	community	[60,70)	[80,Inf]	0.4458528069089014
baseline	community	[70,80)	[0,5)	0.15288264038518534
baseline	community	[70,80)	[5,10)	0.1691882427223992
baseline	community	[70,80)	[10,20)	0.3831874437865965
baseline	community	[70,80)	[20,30)	0.5415955205007799
baseline	community	[70,80)	[30,40)	0.6364866589282963
baseline	community	[70,80)	[40,50)	0.8574226496403199
baseline	community	[70,80)	[50,60)	1.0562487581845932
baseline	community	[70,80)	[60,70)	1.4286036524144088
baseline	community	[70,80)	[70,80)	1.9440214639532774
baseline	community	[70,80)	[80,Inf]	0.8816184691740818
baseline	community	[80,Inf]	[0,5)	0.09703373512796923
baseline	community	[80,Inf]	[5,10)	0.0893831890356755
baseline	community	[80,Inf]	[10,20)	0.2410977108373206
baseline	community	[80,Inf]	[20,30)	0.5930437698335163
baseline	community	[80,Inf]	[30,40)	0.6725632936980316
baseline	community	[80,Inf]	[40,50)	0.7695547307240866
baseline	community	[80,Inf]	[50,60)	1.0770428063123256
baseline	community	[80,Inf]	[60,70)	1.2157222390384788
baseline	community	[80,Inf]	[70,80)	1.5916509629257756
baseline	community	[80,Inf]	[80,Inf]	1.215049971559131
baseline	household	[0,5)	[0,5)	0.4070408625662851
baseline	household	[0,5)	[5,10)	0.32730910398861673
baseline	household	[0,5)	[10,20)	0.10014262765699103
baseline	household	[0,5)	[20,30)	0.24758823467515287
baseline	household	[0,5)	[30,40)	1.061190289703929
baseline	household	[0,5)	[40,50)	0.2608654922377719
baseline	household	[0,5)	[50,60)	0.015498542453068838
baseline	household	[0,5)	[60,70)	0.0021364249791333842
baseline	household	[0,5)	[70,80)	6.746020620367068e-4
baseline	household	[0,5)	[80,Inf]	1.3260271162515738e-4

baseline	household	[5,10)	[0,5)	0.30727042637434937
baseline	household	[5,10)	[5,10)	0.41975894142772285
baseline	household	[5,10)	[10,20)	0.4163466135534939
baseline	household	[5,10)	[20,30)	0.07982204035388576
baseline	household	[5,10)	[30,40)	0.674127507984427
baseline	household	[5,10)	[40,50)	0.7216065752826084
baseline	household	[5,10)	[50,60)	0.0797467087718552
baseline	household	[5,10)	[60,70)	0.009696030256648487
baseline	household	[5,10)	[70,80)	0.0018323544172112478
baseline	household	[5,10)	[80,Inf]	4.828996207068691e-4
baseline	household	[10,20)	[0,5)	0.043334485608693026
baseline	household	[10,20)	[5,10)	0.19192683534893304
baseline	household	[10,20)	[10,20)	0.8575386664602787
baseline	household	[10,20)	[20,30)	0.19114860330716
baseline	household	[10,20)	[30,40)	0.1295715987274422
baseline	household	[10,20)	[40,50)	0.9314284116356336
baseline	household	[10,20)	[50,60)	0.4347193662378137
baseline	household	[10,20)	[60,70)	0.03193287786356278
baseline	household	[10,20)	[70,80)	0.005162709356613486
baseline	household	[10,20)	[80,Inf]	0.002158716603376439
baseline	household	[20,30)	[0,5)	0.10015923985769302
baseline	household	[20,30)	[5,10)	0.034394692173712015
baseline	household	[20,30)	[10,20)	0.17868311108042392
baseline	household	[20,30)	[20,30)	0.5514599619661026
baseline	household	[20,30)	[30,40)	0.09706891014570301
baseline	household	[20,30)	[40,50)	0.10507481518187532
baseline	household	[20,30)	[50,60)	0.38509862675927303
baseline	household	[20,30)	[60,70)	0.09395158793150896
baseline	household	[20,30)	[70,80)	0.007617222139560039
baseline	household	[20,30)	[80,Inf]	0.0020881361139043126
baseline	household	[30,40)	[0,5)	0.45149630793426443

baseline	household	[30,40)	[5,10)	0.30552035871744104
baseline	household	[30,40)	[10,20)	0.12738909728854642
baseline	household	[30,40)	[20,30)	0.10208895446142797
baseline	household	[30,40)	[30,40)	0.5004214984405465
baseline	household	[30,40)	[40,50)	0.13673597023713677
baseline	household	[30,40)	[50,60)	0.03556035266058932
baseline	household	[30,40)	[60,70)	0.0361682660710427
baseline	household	[30,40)	[70,80)	0.006123515348726495
baseline	household	[30,40)	[80,Inf]	0.0027158625499718665
baseline	household	[40,50)	[0,5)	0.09656426743315541
baseline	household	[40,50)	[5,10)	0.28454562000706995
baseline	household	[40,50)	[10,20)	0.796763115027339
baseline	household	[40,50)	[20,30)	0.09615209475220465
baseline	household	[40,50)	[30,40)	0.1189700551974524
baseline	household	[40,50)	[40,50)	0.45569203712092426
baseline	household	[40,50)	[50,60)	0.12059645690895449
baseline	household	[40,50)	[60,70)	0.01681550585088913
baseline	household	[40,50)	[70,80)	0.009172671252767791
baseline	household	[40,50)	[80,Inf]	0.0032941308275034483
baseline	household	[50,60)	[0,5)	0.0054663645330916295
baseline	household	[50,60)	[5,10)	0.029960975606369657
baseline	household	[50,60)	[10,20)	0.3542990349901927
baseline	household	[50,60)	[20,30)	0.3357545322760951
baseline	household	[50,60)	[30,40)	0.02948081306993221
baseline	household	[50,60)	[40,50)	0.1148991237605592
baseline	household	[50,60)	[50,60)	0.47037489993179143
baseline	household	[50,60)	[60,70)	0.11123235392751113
baseline	household	[50,60)	[70,80)	0.00818798645408956
baseline	household	[50,60)	[80,Inf]	0.00370180543914048
baseline	household	[60,70)	[0,5)	8.942349430740162e-4
baseline	household	[60,70)	[5,10)	0.004323055630922437

baseline	household	[60,70)	[10,20)	0.030888665598864433
baseline	household	[60,70)	[20,30)	0.09721867881516932
baseline	household	[60,70)	[30,40)	0.03558499530029821
baseline	household	[60,70)	[40,50)	0.01901412063979772
baseline	household	[60,70)	[50,60)	0.13200664925416708
baseline	household	[60,70)	[60,70)	0.5716054838561209
baseline	household	[60,70)	[70,80)	0.08511377080125268
baseline	household	[60,70)	[80,Inf]	0.0064229007327088325
baseline	household	[70,80)	[0,5)	4.264933009124747e-4
baseline	household	[70,80)	[5,10)	0.001233995620044588
baseline	household	[70,80)	[10,20)	0.007542505097738782
baseline	household	[70,80)	[20,30)	0.011904511253696978
baseline	household	[70,80)	[30,40)	0.009099708802517582
baseline	household	[70,80)	[40,50)	0.01566630621038694
baseline	household	[70,80)	[50,60)	0.014676992488601277
baseline	household	[70,80)	[60,70)	0.12855789720071428
baseline	household	[70,80)	[70,80)	0.5202111855867388
baseline	household	[70,80)	[80,Inf]	0.08363388880376807
baseline	household	[80,Inf]	[0,5)	1.5134316002273797e-4
baseline	household	[80,Inf]	[5,10)	5.870930907831077e-4
baseline	household	[80,Inf]	[10,20)	0.005693467680223771
baseline	household	[80,Inf]	[20,30)	0.005891440235588357
baseline	household	[80,Inf]	[30,40)	0.007285718943480542
baseline	household	[80,Inf]	[40,50)	0.010156714819004676
baseline	household	[80,Inf]	[50,60)	0.011979105642857717
baseline	household	[80,Inf]	[60,70)	0.01751365501393864
baseline	household	[80,Inf]	[70,80)	0.15099598860263677
baseline	household	[80,Inf]	[80,Inf]	0.2683356020398899
April 2020	all	[0,5)	[0,5)	1.1760647800283484
April 2020	all	[0,5)	[5,10)	0.5875298876820264
April 2020	all	[0,5)	[10,20)	0.5400699416922248

April 2020	all	[0,5)	[20,30)	0.578921241529211
April 2020	all	[0,5)	[30,40)	1.021089250584432
April 2020	all	[0,5)	[40,50)	0.7932185991971371
April 2020	all	[0,5)	[50,60)	0.5726735956629352
April 2020	all	[0,5)	[60,70)	0.5126679296879905
April 2020	all	[0,5)	[70,80)	0.11706925879403335
April 2020	all	[0,5)	[80,Inf]	0.017247730603044378
April 2020	all	[5,10)	[0,5)	0.5546231624103924
April 2020	all	[5,10)	[5,10)	1.1257333803029383
April 2020	all	[5,10)	[10,20)	0.9137547883991088
April 2020	all	[5,10)	[20,30)	0.5722564331973451
April 2020	all	[5,10)	[30,40)	0.8518177612831974
April 2020	all	[5,10)	[40,50)	0.9383075401244417
April 2020	all	[5,10)	[50,60)	0.5077781832573309
April 2020	all	[5,10)	[60,70)	0.2443319513726792
April 2020	all	[5,10)	[70,80)	0.08326748033456127
April 2020	all	[5,10)	[80,Inf]	0.01669700083175268
April 2020	all	[10,20)	[0,5)	0.23289665817247532
April 2020	all	[10,20)	[5,10)	0.4174218509556902
April 2020	all	[10,20)	[10,20)	1.5201235614755577
April 2020	all	[10,20)	[20,30)	0.7569827538265377
April 2020	all	[10,20)	[30,40)	0.6665149023664506
April 2020	all	[10,20)	[40,50)	0.9677321210649882
April 2020	all	[10,20)	[50,60)	0.6708700048095472
April 2020	all	[10,20)	[60,70)	0.2217230020923736
April 2020	all	[10,20)	[70,80)	0.0937691622653481
April 2020	all	[10,20)	[80,Inf]	0.026800362071121887
April 2020	all	[20,30)	[0,5)	0.22771692331426435
April 2020	all	[20,30)	[5,10)	0.23844946869220268
April 2020	all	[20,30)	[10,20)	0.6904716699375492
April 2020	all	[20,30)	[20,30)	1.2875902393239995

April 2020	all	[20,30)	[30,40)	0.8736405382064821
April 2020	all	[20,30)	[40,50)	0.8573686311665957
April 2020	all	[20,30)	[50,60)	0.8051095733181638
April 2020	all	[20,30)	[60,70)	0.3091656184405833
April 2020	all	[20,30)	[70,80)	0.12005911840002544
April 2020	all	[20,30)	[80,Inf]	0.052768007370853326
April 2020	all	[30,40)	[0,5)	0.41947505249281714
April 2020	all	[30,40)	[5,10)	0.3706954763928983
April 2020	all	[30,40)	[10,20)	0.6349430796839566
April 2020	all	[30,40)	[20,30)	0.9124227361155588
April 2020	all	[30,40)	[30,40)	1.425624195188558
April 2020	all	[30,40)	[40,50)	1.089056042761873
April 2020	all	[30,40)	[50,60)	0.8073879405068872
April 2020	all	[30,40)	[60,70)	0.38127559978627085
April 2020	all	[30,40)	[70,80)	0.15799781337183094
April 2020	all	[30,40)	[80,Inf]	0.07013617722233088
April 2020	all	[40,50)	[0,5)	0.30382143439951365
April 2020	all	[40,50)	[5,10)	0.3807210421679003
April 2020	all	[40,50)	[10,20)	0.8595528259943088
April 2020	all	[40,50)	[20,30)	0.8348784907688682
April 2020	all	[40,50)	[30,40)	1.0154099812627861
April 2020	all	[40,50)	[40,50)	1.6283033180882782
April 2020	all	[40,50)	[50,60)	0.9292888720601618
April 2020	all	[40,50)	[60,70)	0.3998304551518352
April 2020	all	[40,50)	[70,80)	0.22617403117424129
April 2020	all	[40,50)	[80,Inf]	0.12912370474498536
April 2020	all	[50,60)	[0,5)	0.19772531877870142
April 2020	all	[50,60)	[5,10)	0.18571903159831654
April 2020	all	[50,60)	[10,20)	0.5371248807897762
April 2020	all	[50,60)	[20,30)	0.7067008260093693
April 2020	all	[50,60)	[30,40)	0.6785808236519729

April 2020	all	[50,60)	[40,50)	0.8376816314756509
April 2020	all	[50,60)	[50,60)	1.211741845367384
April 2020	all	[50,60)	[60,70)	0.5426519565509331
April 2020	all	[50,60)	[70,80)	0.3174927722904943
April 2020	all	[50,60)	[80,Inf]	0.18915510017686157
April 2020	all	[60,70)	[0,5)	0.2124389334190808
April 2020	all	[60,70)	[5,10)	0.10725859096261814
April 2020	all	[60,70)	[10,20)	0.21306864265187175
April 2020	all	[60,70)	[20,30)	0.32571362757022626
April 2020	all	[60,70)	[30,40)	0.38460967260228335
April 2020	all	[60,70)	[40,50)	0.4325827300600576
April 2020	all	[60,70)	[50,60)	0.6513061026743447
April 2020	all	[60,70)	[60,70)	0.9218564475858257
April 2020	all	[60,70)	[70,80)	0.44369546062196863
April 2020	all	[60,70)	[80,Inf]	0.1640669735419115
April 2020	all	[70,80)	[0,5)	0.06661145404993946
April 2020	all	[70,80)	[5,10)	0.050189429853607886
April 2020	all	[70,80)	[10,20)	0.12372205137478436
April 2020	all	[70,80)	[20,30)	0.17366779764527143
April 2020	all	[70,80)	[30,40)	0.2188340398270445
April 2020	all	[70,80)	[40,50)	0.33598329258574183
April 2020	all	[70,80)	[50,60)	0.5232065225770091
April 2020	all	[70,80)	[60,70)	0.6092103228919242
April 2020	all	[70,80)	[70,80)	0.9948947652733395
April 2020	all	[70,80)	[80,Inf]	0.19782349750611133
April 2020	all	[80,Inf]	[0,5)	0.018699794982934767
April 2020	all	[80,Inf]	[5,10)	0.019176527275207744
April 2020	all	[80,Inf]	[10,20)	0.06738025628839803
April 2020	all	[80,Inf]	[20,30)	0.14543851451825143
April 2020	all	[80,Inf]	[30,40)	0.18509738898066636
April 2020	all	[80,Inf]	[40,50)	0.36548403715753963

April 2020	all	[80,Inf]	[50,60)	0.5939460521196533
April 2020	all	[80,Inf]	[60,70)	0.42923606352322935
April 2020	all	[80,Inf]	[70,80)	0.37694670495451443
April 2020	all	[80,Inf]	[80,Inf]	0.3621969070338579
April 2020	community	[0,5)	[0,5)	0.3646066937544967
April 2020	community	[0,5)	[5,10)	0.23277438675886775
April 2020	community	[0,5)	[10,20)	0.24736496258080135
April 2020	community	[0,5)	[20,30)	0.18699000414917533
April 2020	community	[0,5)	[30,40)	0.21353930869007756
April 2020	community	[0,5)	[40,50)	0.2669119038322729
April 2020	community	[0,5)	[50,60)	0.36745877473361
April 2020	community	[0,5)	[60,70)	0.43059228077196454
April 2020	community	[0,5)	[70,80)	0.07987992594629864
April 2020	community	[0,5)	[80,Inf]	0.007390587548313975
April 2020	community	[5,10)	[0,5)	0.2197370975636243
April 2020	community	[5,10)	[5,10)	0.683453529241781
April 2020	community	[5,10)	[10,20)	0.49385228854092655
April 2020	community	[5,10)	[20,30)	0.26357825712998617
April 2020	community	[5,10)	[30,40)	0.26349264363918223
April 2020	community	[5,10)	[40,50)	0.29182560088503934
April 2020	community	[5,10)	[50,60)	0.24031403100412996
April 2020	community	[5,10)	[60,70)	0.1782300654192099
April 2020	community	[5,10)	[70,80)	0.069685130524172
April 2020	community	[5,10)	[80,Inf]	0.013337025180202581
April 2020	community	[10,20)	[0,5)	0.10667149707413237
April 2020	community	[10,20)	[5,10)	0.2256043219871066
April 2020	community	[10,20)	[10,20)	0.9032642036534505
April 2020	community	[10,20)	[20,30)	0.4483058240342007
April 2020	community	[10,20)	[30,40)	0.3291359832490927
April 2020	community	[10,20)	[40,50)	0.34092990591315925
April 2020	community	[10,20)	[50,60)	0.26477395554638056

April 2020	community	[10,20)	[60,70)	0.14451851163532395
April 2020	community	[10,20)	[70,80)	0.08441104043783408
April 2020	community	[10,20)	[80,Inf]	0.027938710374217076
April 2020	community	[20,30)	[0,5)	0.07355077851855162
April 2020	community	[20,30)	[5,10)	0.10982892329353772
April 2020	community	[20,30)	[10,20)	0.40891655512922537
April 2020	community	[20,30)	[20,30)	0.8663049896974029
April 2020	community	[20,30)	[30,40)	0.5976289047242478
April 2020	community	[20,30)	[40,50)	0.5398641140140658
April 2020	community	[20,30)	[50,60)	0.45809694574239823
April 2020	community	[20,30)	[60,70)	0.21149913300220144
April 2020	community	[20,30)	[70,80)	0.10860624025236812
April 2020	community	[20,30)	[80,Inf]	0.055586327309509215
April 2020	community	[30,40)	[0,5)	0.08772498514311655
April 2020	community	[30,40)	[5,10)	0.11466784587421879
April 2020	community	[30,40)	[10,20)	0.3135434437330058
April 2020	community	[30,40)	[20,30)	0.6241601718961317
April 2020	community	[30,40)	[30,40)	1.0500851417157624
April 2020	community	[30,40)	[40,50)	0.8709354947403554
April 2020	community	[30,40)	[50,60)	0.6696360655506691
April 2020	community	[30,40)	[60,70)	0.32819652583905246
April 2020	community	[30,40)	[70,80)	0.14990549735872585
April 2020	community	[30,40)	[80,Inf]	0.07375344392677897
April 2020	community	[40,50)	[0,5)	0.10223300125976902
April 2020	community	[40,50)	[5,10)	0.11841096546566689
April 2020	community	[40,50)	[10,20)	0.3028187536660655
April 2020	community	[40,50)	[20,30)	0.5257035167693755
April 2020	community	[40,50)	[30,40)	0.8120403436855991
April 2020	community	[40,50)	[40,50)	1.2813294884896065
April 2020	community	[40,50)	[50,60)	0.7724308882129276
April 2020	community	[40,50)	[60,70)	0.3596952451612189

April 2020	community	[40,50)	[70,80)	0.21796061827260754
April 2020	community	[40,50)	[80,Inf]	0.13296213008975902
April 2020	community	[50,60)	[0,5)	0.1268710436554203
April 2020	community	[50,60)	[5,10)	0.08789438479384616
April 2020	community	[50,60)	[10,20)	0.21198821941478735
April 2020	community	[50,60)	[20,30)	0.4021032683091342
April 2020	community	[50,60)	[30,40)	0.5628062682149229
April 2020	community	[50,60)	[40,50)	0.6962880057608908
April 2020	community	[50,60)	[50,60)	0.8022038630828249
April 2020	community	[50,60)	[60,70)	0.3935406785936754
April 2020	community	[50,60)	[70,80)	0.2688530227728949
April 2020	community	[50,60)	[80,Inf]	0.18498821969097723
April 2020	community	[60,70)	[0,5)	0.17842600120833832
April 2020	community	[60,70)	[5,10)	0.07824122707796444
April 2020	community	[60,70)	[10,20)	0.13887774327995553
April 2020	community	[60,70)	[20,30)	0.22282027848282016
April 2020	community	[60,70)	[30,40)	0.33106598446630253
April 2020	community	[60,70)	[40,50)	0.38916007761599136
April 2020	community	[60,70)	[50,60)	0.47233795039278476
April 2020	community	[60,70)	[60,70)	0.4661455597492005
April 2020	community	[60,70)	[70,80)	0.2704888180414184
April 2020	community	[60,70)	[80,Inf]	0.12028937093965515
April 2020	community	[70,80)	[0,5)	0.04545065393408422
April 2020	community	[70,80)	[5,10)	0.04200266726515043
April 2020	community	[70,80)	[10,20)	0.1113744583959231
April 2020	community	[70,80)	[20,30)	0.15710081459804948
April 2020	community	[70,80)	[30,40)	0.20762584773598786
April 2020	community	[70,80)	[40,50)	0.3237822695330862
April 2020	community	[70,80)	[50,60)	0.44304954538274294
April 2020	community	[70,80)	[60,70)	0.3713921209855806
April 2020	community	[70,80)	[70,80)	0.47596126690675467

April 2020	community	[70,80)	[80,Inf]	0.06728406924411334
April 2020	community	[80,Inf]	[0,5)	0.008012703461671539
April 2020	community	[80,Inf]	[5,10)	0.015317583313786012
April 2020	community	[80,Inf]	[10,20)	0.07024226973952001
April 2020	community	[80,Inf]	[20,30)	0.15320643293028832
April 2020	community	[80,Inf]	[30,40)	0.19464382061866048
April 2020	community	[80,Inf]	[40,50)	0.3763487908008097
April 2020	community	[80,Inf]	[50,60)	0.5808606610759302
April 2020	community	[80,Inf]	[60,70)	0.31470264600095305
April 2020	community	[80,Inf]	[70,80)	0.12820567013034773
April 2020	community	[80,Inf]	[80,Inf]	0.05645125537473782
April 2020	household	[0,5)	[0,5)	0.8210030831297492
April 2020	household	[0,5)	[5,10)	0.42306356931328293
April 2020	household	[0,5)	[10,20)	0.1682471989405442
April 2020	household	[0,5)	[20,30)	0.2735336221937439
April 2020	household	[0,5)	[30,40)	1.3472612281921932
April 2020	household	[0,5)	[40,50)	0.42903744813946376
April 2020	household	[0,5)	[50,60)	0.06824433269517892
April 2020	household	[0,5)	[60,70)	0.04925730633764947
April 2020	household	[0,5)	[70,80)	0.041633768827222396
April 2020	household	[0,5)	[80,Inf]	0.022796126445601715
April 2020	household	[5,10)	[0,5)	0.3993659450672259
April 2020	household	[5,10)	[5,10)	0.5535338802174224
April 2020	household	[5,10)	[10,20)	0.38419010213155086
April 2020	household	[5,10)	[20,30)	0.08206546677197935
April 2020	household	[5,10)	[30,40)	0.8113877549661407
April 2020	household	[5,10)	[40,50)	0.8760493820565902
April 2020	household	[5,10)	[50,60)	0.10850698668372574
April 2020	household	[5,10)	[60,70)	0.025856832207633084
April 2020	household	[5,10)	[70,80)	0.005871734528101912
April 2020	household	[5,10)	[80,Inf]	0.0011714474677208984

April 2020	household	[10,20)	[0,5)	0.07255534783144396
April 2020	household	[10,20)	[5,10)	0.17550167721775356
April 2020	household	[10,20)	[10,20)	0.9133707082748583
April 2020	household	[10,20)	[20,30)	0.16462111404430427
April 2020	household	[10,20)	[30,40)	0.1466622958513173
April 2020	household	[10,20)	[40,50)	0.9787940977088019
April 2020	household	[10,20)	[50,60)	0.5825898266179115
April 2020	household	[10,20)	[60,70)	0.033802583705954466
April 2020	household	[10,20)	[70,80)	0.002962835771177684
April 2020	household	[10,20)	[80,Inf]	2.2926191625413092e-4
April 2020	household	[20,30)	[0,5)	0.10759750420381252
April 2020	household	[20,30)	[5,10)	0.034195693803276986
April 2020	household	[20,30)	[10,20)	0.15015512783803772
April 2020	household	[20,30)	[20,30)	0.6059278618419952
April 2020	household	[20,30)	[30,40)	0.13651646835073375
April 2020	household	[20,30)	[40,50)	0.08843659722274506
April 2020	household	[20,30)	[50,60)	0.45164119203773445
April 2020	household	[20,30)	[60,70)	0.1139080315556362
April 2020	household	[20,30)	[70,80)	0.00559125105771212
April 2020	household	[20,30)	[80,Inf]	1.8382663901782037e-4
April 2020	household	[30,40)	[0,5)	0.5534718605743049
April 2020	household	[30,40)	[5,10)	0.3531021476706615
April 2020	household	[30,40)	[10,20)	0.1397204408746677
April 2020	household	[30,40)	[20,30)	0.1425717023216554
April 2020	household	[30,40)	[30,40)	0.5666714913088629
April 2020	household	[30,40)	[40,50)	0.13383396773402134
April 2020	household	[30,40)	[50,60)	0.03664159291363168
April 2020	household	[30,40)	[60,70)	0.03463502883356748
April 2020	household	[30,40)	[70,80)	0.008102067845259095
April 2020	household	[30,40)	[80,Inf]	3.9602314900321115e-4
April 2020	household	[40,50)	[0,5)	0.16433111725182115

April 2020	household	[40,50)	[5,10)	0.35545785843375327
April 2020	household	[40,50)	[10,20)	0.8693822992585897
April 2020	household	[40,50)	[20,30)	0.08611676538406984
April 2020	household	[40,50)	[30,40)	0.12478249121147973
April 2020	household	[40,50)	[40,50)	0.5369036489269697
April 2020	household	[40,50)	[50,60)	0.13763134683303116
April 2020	household	[40,50)	[60,70)	0.01901461066032581
April 2020	household	[40,50)	[70,80)	0.005462305131402382
April 2020	household	[40,50)	[80,Inf]	9.604859064088381e-4
April 2020	household	[50,60)	[0,5)	0.023562978244949503
April 2020	household	[50,60)	[5,10)	0.03968588777054593
April 2020	household	[50,60)	[10,20)	0.4664400470802419
April 2020	household	[50,60)	[20,30)	0.3964378012966417
April 2020	household	[50,60)	[30,40)	0.03079551499862011
April 2020	household	[50,60)	[40,50)	0.12406122931490343
April 2020	household	[50,60)	[50,60)	0.5886824544356134
April 2020	household	[50,60)	[60,70)	0.1282062604850048
April 2020	household	[50,60)	[70,80)	0.014572240185588763
April 2020	household	[50,60)	[80,Inf]	0.001552680848167772
April 2020	household	[60,70)	[0,5)	0.020412745714805794
April 2020	household	[60,70)	[5,10)	0.011350436230677942
April 2020	household	[60,70)	[10,20)	0.032483178960315336
April 2020	household	[60,70)	[20,30)	0.12000235668625572
April 2020	household	[60,70)	[30,40)	0.03493826198883607
April 2020	household	[60,70)	[40,50)	0.020571954975940686
April 2020	household	[60,70)	[50,60)	0.1538760732279695
April 2020	household	[60,70)	[60,70)	0.5856426421271737
April 2020	household	[60,70)	[70,80)	0.14207461107184108
April 2020	household	[60,70)	[80,Inf]	0.01136886715725993
April 2020	household	[70,80)	[0,5)	0.023689603156840218
April 2020	household	[70,80)	[5,10)	0.003539171181828776

April 2020	household	[70,80)	[10,20)	0.003909307189095138
April 2020	household	[70,80)	[20,30)	0.00808790942444936
April 2020	household	[70,80)	[30,40)	0.011221697429951017
April 2020	household	[70,80)	[40,50)	0.00811426572720439
April 2020	household	[70,80)	[50,60)	0.02401440795832354
April 2020	household	[70,80)	[60,70)	0.19507147653006224
April 2020	household	[70,80)	[70,80)	0.6283931567250657
April 2020	household	[70,80)	[80,Inf]	0.102833114207292
April 2020	household	[80,Inf]	[0,5)	0.024715327921392898
April 2020	household	[80,Inf]	[5,10)	0.0013454031315745638
April 2020	household	[80,Inf]	[10,20)	5.763932199329193e-4
April 2020	household	[80,Inf]	[20,30)	5.066813799461733e-4
April 2020	household	[80,Inf]	[30,40)	0.0010451609383005439
April 2020	household	[80,Inf]	[40,50)	0.0027186958425426
April 2020	household	[80,Inf]	[50,60)	0.004875603573032729
April 2020	household	[80,Inf]	[60,70)	0.02974381317748782
April 2020	household	[80,Inf]	[70,80)	0.1959534116359983
April 2020	household	[80,Inf]	[80,Inf]	0.39021824450555115
June 2020	all	[0,5)	[0,5)	9.535680974897533
June 2020	all	[0,5)	[5,10)	2.894582679702587
June 2020	all	[0,5)	[10,20)	1.096085602001159
June 2020	all	[0,5)	[20,30)	1.4111914326596
June 2020	all	[0,5)	[30,40)	2.698977139974758
June 2020	all	[0,5)	[40,50)	1.3054750835522104
June 2020	all	[0,5)	[50,60)	0.6310028462405055
June 2020	all	[0,5)	[60,70)	0.49836716906255313
June 2020	all	[0,5)	[70,80)	0.16162444098120982
June 2020	all	[0,5)	[80,Inf]	0.04205784904241579
June 2020	all	[5,10)	[0,5)	2.732347013939343
June 2020	all	[5,10)	[5,10)	15.528862822872497
June 2020	all	[5,10)	[10,20)	3.159605735522081

June 2020	all	[5,10)	[20,30)	1.2030425459587817
June 2020	all	[5,10)	[30,40)	2.035732909205147
June 2020	all	[5,10)	[40,50)	2.2696717834830213
June 2020	all	[5,10)	[50,60)	0.9093114947519015
June 2020	all	[5,10)	[60,70)	0.541291670428993
June 2020	all	[5,10)	[70,80)	0.18942044691607662
June 2020	all	[5,10)	[80,Inf]	0.055314023756475164
June 2020	all	[10,20)	[0,5)	0.4726642589043966
June 2020	all	[10,20)	[5,10)	1.443385263474447
June 2020	all	[10,20)	[10,20)	7.964256213631935
June 2020	all	[10,20)	[20,30)	1.6151615082436357
June 2020	all	[10,20)	[30,40)	1.197062810261488
June 2020	all	[10,20)	[40,50)	1.852809467071242
June 2020	all	[10,20)	[50,60)	1.1666745111896015
June 2020	all	[10,20)	[60,70)	0.48001511558330545
June 2020	all	[10,20)	[70,80)	0.20982790841318677
June 2020	all	[10,20)	[80,Inf]	0.08938818131364801
June 2020	all	[20,30)	[0,5)	0.5551060073638401
June 2020	all	[20,30)	[5,10)	0.5012947475730598
June 2020	all	[20,30)	[10,20)	1.4732527128605506
June 2020	all	[20,30)	[20,30)	3.188285846053175
June 2020	all	[20,30)	[30,40)	1.6199369002308959
June 2020	all	[20,30)	[40,50)	1.3448856447671544
June 2020	all	[20,30)	[50,60)	1.4126696488488917
June 2020	all	[20,30)	[60,70)	0.6096319458853104
June 2020	all	[20,30)	[70,80)	0.23300412362443584
June 2020	all	[20,30)	[80,Inf]	0.11007079794936349
June 2020	all	[30,40)	[0,5)	1.1087512596362359
June 2020	all	[30,40)	[5,10)	0.8859086899089883
June 2020	all	[30,40)	[10,20)	1.140363206107038
June 2020	all	[30,40)	[20,30)	1.691841692771972

June 2020	all	[30,40)	[30,40)	2.75150663849892
June 2020	all	[30,40)	[40,50)	1.7003355955076498
June 2020	all	[30,40)	[50,60)	1.268322998281017
June 2020	all	[30,40)	[60,70)	0.7822809081583649
June 2020	all	[30,40)	[70,80)	0.3012291744889573
June 2020	all	[30,40)	[80,Inf]	0.15308139703338147
June 2020	all	[40,50)	[0,5)	0.5000330521097623
June 2020	all	[40,50)	[5,10)	0.9209339942108333
June 2020	all	[40,50)	[10,20)	1.645681760492174
June 2020	all	[40,50)	[20,30)	1.3096108254278487
June 2020	all	[40,50)	[30,40)	1.5853603639821383
June 2020	all	[40,50)	[40,50)	2.5923077192173163
June 2020	all	[40,50)	[50,60)	1.3773796300369832
June 2020	all	[40,50)	[60,70)	0.694376674930878
June 2020	all	[40,50)	[70,80)	0.3725680259265217
June 2020	all	[40,50)	[80,Inf]	0.17585206637704312
June 2020	all	[50,60)	[0,5)	0.2178708181187665
June 2020	all	[50,60)	[5,10)	0.3325831751572846
June 2020	all	[50,60)	[10,20)	0.9340960678768528
June 2020	all	[50,60)	[20,30)	1.2400088728671457
June 2020	all	[50,60)	[30,40)	1.065988858717057
June 2020	all	[50,60)	[40,50)	1.241607474841456
June 2020	all	[50,60)	[50,60)	1.9099162834389065
June 2020	all	[50,60)	[60,70)	0.7868876413180401
June 2020	all	[50,60)	[70,80)	0.3618812636579212
June 2020	all	[50,60)	[80,Inf]	0.2759041618334919
June 2020	all	[60,70)	[0,5)	0.20652214757392387
June 2020	all	[60,70)	[5,10)	0.23762562647210783
June 2020	all	[60,70)	[10,20)	0.461282107282825
June 2020	all	[60,70)	[20,30)	0.6422717955751382
June 2020	all	[60,70)	[30,40)	0.7891403878043921

June 2020	all	[60,70)	[40,50)	0.7512643140495083	
June 2020	all	[60,70)	[50,60)	0.944472593030437	
June 2020	all	[60,70)	[60,70)	1.322817791275073	
June 2020	all	[60,70)	[70,80)	0.49975606347975243	
June 2020	all	[60,70)	[80,Inf]	0.22743473733403602	
June 2020	all	[70,80)	[0,5)	0.09196213298673543	
June 2020	all	[70,80)	[5,10)	0.11416904467532751	
June 2020	all	[70,80)	[10,20)	0.2768469419501834	
June 2020	all	[70,80)	[20,30)	0.33703762524092934	
June 2020	all	[70,80)	[30,40)	0.4172106756852329	
June 2020	all	[70,80)	[40,50)	0.5534424950319953	
June 2020	all	[70,80)	[50,60)	0.5963616584451491	
June 2020	all	[70,80)	[60,70)	0.686188421177565	
June 2020	all	[70,80)	[70,80)	1.053757211029444	
June 2020	all	[70,80)	[80,Inf]	0.30884140507172686	
June 2020	all	[80,Inf]	[0,5)	0.04559929126211945	
June 2020	all	[80,Inf]	[5,10)	0.06352888360302915	
June 2020	all	[80,Inf]	[10,20)	0.22472771076635492	
June 2020	all	[80,Inf]	[20,30)	0.30338073499752505	
June 2020	all	[80,Inf]	[30,40)	0.4039750457344324	
June 2020	all	[80,Inf]	[40,50)	0.49775139149445113	
June 2020	all	[80,Inf]	[50,60)	0.866347494059004	
June 2020	all	[80,Inf]	[60,70)	0.5950196656811426	
June 2020	all	[80,Inf]	[70,80)	0.5885083175636722	
June 2020	all	[80,Inf]	[80,Inf]	0.6780733075535321	
June 2020	community		[0,5)	[0,5)	8.775098872842303
June 2020	community		[0,5)	[5,10)	2.4259702353676738
June 2020	community		[0,5)	[10,20)	1.1103833392202864
June 2020	community		[0,5)	[20,30)	1.1538423836197214
June 2020	community		[0,5)	[30,40)	1.5190759399490503
June 2020	community		[0,5)	[40,50)	0.951955196484968

June 2020	community	[0,5)	[50,60)	0.6249379087649379
June 2020	community	[0,5)	[60,70)	0.4666778989853839
June 2020	community	[0,5)	[70,80)	0.14869475437014731
June 2020	community	[0,5)	[80,Inf]	0.03379425288270739
June 2020	community	[5,10)	[0,5)	2.290001145338284
June 2020	community	[5,10)	[5,10)	13.552049152764543
June 2020	community	[5,10)	[10,20)	2.9356077086962933
June 2020	community	[5,10)	[20,30)	1.1423310980000407
June 2020	community	[5,10)	[30,40)	1.2885070984745541
June 2020	community	[5,10)	[40,50)	1.3568846710655844
June 2020	community	[5,10)	[50,60)	0.7538392112214657
June 2020	community	[5,10)	[60,70)	0.4908710905316382
June 2020	community	[5,10)	[70,80)	0.18686255277292846
June 2020	community	[5,10)	[80,Inf]	0.05401180060028314
June 2020	community	[10,20)	[0,5)	0.47883012390150836
June 2020	community	[10,20)	[5,10)	1.3410573634296992
June 2020	community	[10,20)	[10,20)	6.773912647595956
June 2020	community	[10,20)	[20,30)	1.537014201167461
June 2020	community	[10,20)	[30,40)	0.9747800048936158
June 2020	community	[10,20)	[40,50)	1.022910795326035
June 2020	community	[10,20)	[50,60)	0.7384084271672123
June 2020	community	[10,20)	[60,70)	0.426729596102731
June 2020	community	[10,20)	[70,80)	0.21008282515054444
June 2020	community	[10,20)	[80,Inf]	0.0878646652867293
June 2020	community	[20,30)	[0,5)	0.45387279023632754
June 2020	community	[20,30)	[5,10)	0.47599607292386387
June 2020	community	[20,30)	[10,20)	1.4019729473041542
June 2020	community	[20,30)	[20,30)	2.5316710137865135
June 2020	community	[20,30)	[30,40)	1.4316037237039811
June 2020	community	[20,30)	[40,50)	1.1217014761080693
June 2020	community	[20,30)	[50,60)	1.0102189739176024

June 2020	community	[20,30)	[60,70)	0.5159904157884702
June 2020	community	[20,30)	[70,80)	0.23588225957257405
June 2020	community	[20,30)	[80,Inf]	0.11108293014612677
June 2020	community	[30,40)	[0,5)	0.6240403786736254
June 2020	community	[30,40)	[5,10)	0.5607293018946535
June 2020	community	[30,40)	[10,20)	0.9286071773281189
June 2020	community	[30,40)	[20,30)	1.4951520950374677
June 2020	community	[30,40)	[30,40)	2.2311298092518848
June 2020	community	[30,40)	[40,50)	1.5319243947719958
June 2020	community	[30,40)	[50,60)	1.1771962014747186
June 2020	community	[30,40)	[60,70)	0.7120570167812902
June 2020	community	[30,40)	[70,80)	0.30426648426527575
June 2020	community	[30,40)	[80,Inf]	0.15006596144293008
June 2020	community	[40,50)	[0,5)	0.3646263017982732
June 2020	community	[40,50)	[5,10)	0.5505709222617169
June 2020	community	[40,50)	[10,20)	0.9085591853050433
June 2020	community	[40,50)	[20,30)	1.0922795965796408
June 2020	community	[40,50)	[30,40)	1.428338470494126
June 2020	community	[40,50)	[40,50)	2.0961542356708267
June 2020	community	[40,50)	[50,60)	1.2482588853473104
June 2020	community	[40,50)	[60,70)	0.6716206564958566
June 2020	community	[40,50)	[70,80)	0.36804094486789013
June 2020	community	[40,50)	[80,Inf]	0.17889320036735087
June 2020	community	[50,60)	[0,5)	0.2157755360585036
June 2020	community	[50,60)	[5,10)	0.27571922499306384
June 2020	community	[50,60)	[10,20)	0.5912084956184512
June 2020	community	[50,60)	[20,30)	0.8867483185656975
June 2020	community	[50,60)	[30,40)	0.9893992050106263
June 2020	community	[50,60)	[40,50)	1.1252170152945618
June 2020	community	[50,60)	[50,60)	1.3862481174634778
June 2020	community	[50,60)	[60,70)	0.6629574060044834

June 2020	community	[50,60)	[70,80)	0.3530105968618785
June 2020	community	[50,60)	[80,Inf]	0.25740753477751316
June 2020	community	[60,70)	[0,5)	0.1933898601910007
June 2020	community	[60,70)	[5,10)	0.21549109039089567
June 2020	community	[60,70)	[10,20)	0.41007603386055513
June 2020	community	[60,70)	[20,30)	0.5436182555117914
June 2020	community	[60,70)	[30,40)	0.7183002163729504
June 2020	community	[60,70)	[40,50)	0.7266436421942291
June 2020	community	[60,70)	[50,60)	0.7957274648405908
June 2020	community	[60,70)	[60,70)	0.7985840451853425
June 2020	community	[60,70)	[70,80)	0.38084484648204153
June 2020	community	[60,70)	[80,Inf]	0.20961158013602318
June 2020	community	[70,80)	[0,5)	0.0846047255900723
June 2020	community	[70,80)	[5,10)	0.1126275038869663
June 2020	community	[70,80)	[10,20)	0.27718379452992353
June 2020	community	[70,80)	[20,30)	0.3412021585774272
June 2020	community	[70,80)	[30,40)	0.42141843199238316
June 2020	community	[70,80)	[40,50)	0.5467180734153767
June 2020	community	[70,80)	[50,60)	0.5817443599190796
June 2020	community	[70,80)	[60,70)	0.5229189505913454
June 2020	community	[70,80)	[70,80)	0.5314860041145427
June 2020	community	[70,80)	[80,Inf]	0.21658469478934658
June 2020	community	[80,Inf]	[0,5)	0.0366395209554553
June 2020	community	[80,Inf]	[5,10)	0.06203320906088339
June 2020	community	[80,Inf]	[10,20)	0.22089802053213695
June 2020	community	[80,Inf]	[20,30)	0.3061711094074914
June 2020	community	[80,Inf]	[30,40)	0.3960194587411256
June 2020	community	[80,Inf]	[40,50)	0.5063603581546733
June 2020	community	[80,Inf]	[50,60)	0.8082681529701518
June 2020	community	[80,Inf]	[60,70)	0.5483905508804039
June 2020	community	[80,Inf]	[70,80)	0.41270721302576

June 2020	community	[80,Inf]	[80,Inf]	0.3375901128630202
June 2020	household	[0,5)	[0,5)	0.5210517837663807
June 2020	household	[0,5)	[5,10)	0.47161796656479554
June 2020	household	[0,5)	[10,20)	0.1129612170885154
June 2020	household	[0,5)	[20,30)	0.2110650531583942
June 2020	household	[0,5)	[30,40)	1.40442523338465
June 2020	household	[0,5)	[40,50)	0.35211564021871034
June 2020	household	[0,5)	[50,60)	0.040395773902615104
June 2020	household	[0,5)	[60,70)	0.01838517853482463
June 2020	household	[0,5)	[70,80)	0.01920090205432248
June 2020	household	[0,5)	[80,Inf]	0.010207703649229514
June 2020	household	[5,10)	[0,5)	0.44518958587836016
June 2020	household	[5,10)	[5,10)	0.46955233078719194
June 2020	household	[5,10)	[10,20)	0.461260651149731
June 2020	household	[5,10)	[20,30)	0.0518958298687116
June 2020	household	[5,10)	[30,40)	0.8439388876357736
June 2020	household	[5,10)	[40,50)	0.9544161861328387
June 2020	household	[5,10)	[50,60)	0.09034766603430291
June 2020	household	[5,10)	[60,70)	0.01680190754135318
June 2020	household	[5,10)	[70,80)	0.0031782837866304726
June 2020	household	[5,10)	[80,Inf]	9.521917498046157e-4
June 2020	household	[10,20)	[0,5)	0.048712927495235925
June 2020	household	[10,20)	[5,10)	0.21071106813379206
June 2020	household	[10,20)	[10,20)	0.9079404680065828
June 2020	household	[10,20)	[20,30)	0.18368516744792399
June 2020	household	[10,20)	[30,40)	0.17952547604305386
June 2020	household	[10,20)	[40,50)	1.078992973800357
June 2020	household	[10,20)	[50,60)	0.5070614059123068
June 2020	household	[10,20)	[60,70)	0.03540529645931906
June 2020	household	[10,20)	[70,80)	0.004266861216366651
June 2020	household	[10,20)	[80,Inf]	4.1207697013029244e-4

June 2020	household	[20,30)	[0,5)	0.08302731074577999
June 2020	household	[20,30)	[5,10)	0.021624385881006722
June 2020	household	[20,30)	[10,20)	0.16754336505238432
June 2020	household	[20,30)	[20,30)	0.7436410356844917
June 2020	household	[20,30)	[30,40)	0.12702788473097879
June 2020	household	[20,30)	[40,50)	0.09673725222325548
June 2020	household	[20,30)	[50,60)	0.43227893733687317
June 2020	household	[20,30)	[60,70)	0.10280266382733491
June 2020	household	[20,30)	[70,80)	0.004879997797658274
June 2020	household	[20,30)	[80,Inf]	1.89706508547085e-4
June 2020	household	[30,40)	[0,5)	0.5769477291098235
June 2020	household	[30,40)	[5,10)	0.36726894515968267
June 2020	household	[30,40)	[10,20)	0.17102521576679017
June 2020	household	[30,40)	[20,30)	0.13266133158992985
June 2020	household	[30,40)	[30,40)	0.5888146667113131
June 2020	household	[30,40)	[40,50)	0.14331295179462222
June 2020	household	[30,40)	[50,60)	0.03436917073417894
June 2020	household	[30,40)	[60,70)	0.03278334699342645
June 2020	household	[30,40)	[70,80)	0.006535719213940716
June 2020	household	[30,40)	[80,Inf]	2.799029537185296e-4
June 2020	household	[40,50)	[0,5)	0.1348679810619103
June 2020	household	[40,50)	[5,10)	0.3872513797302199
June 2020	household	[40,50)	[10,20)	0.9583696874909226
June 2020	household	[40,50)	[20,30)	0.09420174172925586
June 2020	household	[40,50)	[30,40)	0.1336188708348136
June 2020	household	[40,50)	[40,50)	0.5594037917328785
June 2020	household	[40,50)	[50,60)	0.1331505637534002
June 2020	household	[40,50)	[60,70)	0.0168214571202433
June 2020	household	[40,50)	[70,80)	0.009169406805725788
June 2020	household	[40,50)	[80,Inf]	9.877261879380193e-4
June 2020	household	[50,60)	[0,5)	0.013947731479694091

June 2020	household	[50,60)	[5,10)	0.03304426671858822
June 2020	household	[50,60)	[10,20)	0.405972362719836
June 2020	household	[50,60)	[20,30)	0.3794423209791157
June 2020	household	[50,60)	[30,40)	0.02888572507899006
June 2020	household	[50,60)	[40,50)	0.1200199846023595
June 2020	household	[50,60)	[50,60)	0.5689734916991416
June 2020	household	[50,60)	[60,70)	0.12410912094062025
June 2020	household	[50,60)	[70,80)	0.01352576816704563
June 2020	household	[50,60)	[80,Inf]	0.004099439958106652
June 2020	household	[60,70)	[0,5)	0.007619015192992298
June 2020	household	[60,70)	[5,10)	0.007375818474457062
June 2020	household	[60,70)	[10,20)	0.0340234003943412
June 2020	household	[60,70)	[20,30)	0.10830363349883572
June 2020	household	[60,70)	[30,40)	0.033070751727130365
June 2020	household	[60,70)	[40,50)	0.018199162736442124
June 2020	household	[60,70)	[50,60)	0.1489557513295358
June 2020	household	[60,70)	[60,70)	0.5812627518383996
June 2020	household	[60,70)	[70,80)	0.12447169887151541
June 2020	household	[60,70)	[80,Inf]	0.0108029953652614
June 2020	household	[70,80)	[0,5)	0.010925404866807157
June 2020	household	[70,80)	[5,10)	0.001915695273840959
June 2020	household	[70,80)	[10,20)	0.00562998679034384
June 2020	household	[70,80)	[20,30)	0.0070590232868419775
June 2020	household	[70,80)	[30,40)	0.009052133387088662
June 2020	household	[70,80)	[40,50)	0.013621545105182122
June 2020	household	[70,80)	[50,60)	0.0222893676317624
June 2020	household	[70,80)	[60,70)	0.17090334270844273
June 2020	household	[70,80)	[70,80)	0.5640146532863117
June 2020	household	[70,80)	[80,Inf]	0.08974472980326166
June 2020	household	[80,Inf]	[0,5)	0.011067131478208
June 2020	household	[80,Inf]	[5,10)	0.0010935890305874636

June 2020	household	[80,Inf]	[10,20)	0.0010360070376604401
June 2020	household	[80,Inf]	[20,30)	5.228881272695691e-4
June 2020	household	[80,Inf]	[30,40)	7.387035450784288e-4
June 2020	household	[80,Inf]	[40,50)	0.002795805330291876
June 2020	household	[80,Inf]	[50,60)	0.012872684713738354
June 2020	household	[80,Inf]	[60,70)	0.02826330604183391
June 2020	household	[80,Inf]	[70,80)	0.17101566452288947
June 2020	household	[80,Inf]	[80,Inf]	0.3589858536773081
